# C-terminus Proteolysis and Palmitoylation Cooperate for Optimal Plasma Membrane Localization of RasA in *Aspergillus fumigatus*

**DOI:** 10.3389/fmicb.2018.00562

**Published:** 2018-03-26

**Authors:** Qusai Al Abdallah, Adela Martin-Vicente, Ana Camila Oliveira Souza, Wenbo Ge, Jarrod R. Fortwendel

**Affiliations:** Department of Clinical Pharmacy and Translational Science, University of Tennessee Health Science Center, Memphis, TN, United States

**Keywords:** *Aspergillus*, Ras, CAAX, prenylation, palmitoylation, cell wall

## Abstract

RasA is a major regulator of fungal morphogenesis and virulence in *Aspergillus fumigatus*. The proper localization of RasA to the plasma membrane is essential for the formation of invasive hyphae during infection. In yeast, the localization of Ras2p to the plasma membrane is orchestrated by several post-translational modifications (PTM) at the C-terminal CAAX box that are thought to occur in sequential order. These PTMs include: (1) CAAX motif farnesylation by the farnesyltransferase complex composed of Ram1p and Ram2p; (2) proteolysis of the -AAX residues by Rce1p or Ste24p; (3) methylation of the remaining prenylated cysteine residue by Ste14p, and; (4) palmitoylation at a single conserved cysteine residue mediated by the Erf2p/Erf4p palmitoyltransferase. We previously reported that homologs of each RasA PTM enzyme are conserved in *A. fumigatus*. Additionally, we delineated a major role for protein farnesylation in *A. fumigatus* growth and virulence. In this work, we characterize the post-prenylation processing enzymes of RasA in *A. fumigatus*. The genes encoding the RasA post-prenylation enzymes were first deleted and examined for their roles in growth and regulation of RasA. Only when strains lacked *cppB*, the *A. fumigatus* homologue of yeast *RCE1*, there was a significant reduction in fungal growth and conidial germination. In addition, *cppB*-deletion mutants displayed hypersensitivity to the cell wall-perturbing agents Calcofluor White and Congo Red and the cell wall biosynthesis inhibitor Caspofungin. In contrast to the previously published data in yeast, the deletion of post-prenylation modifying enzymes did not alter the plasma membrane localization or activation of RasA. To delineate the molecular mechanisms underlying these differences, we investigated the interplay between dual-palmitoylation of the RasA hypervariable region and CAAX proteolysis for stabilization of RasA at the plasma membrane. Our data indicate that, in the absence of proper CAAX proteolysis, RasA accumulation at the plasma membrane is stabilized by dual palmitoyl groups on the dual cysteine residues. Therefore, we conclude CAAX proteolysis and dual-palmitoylation of the hypervariable region is important for maintaining a stable attachment association of RasA with the plasma membrane to support optimal fungal growth and development.

## Introduction

Ras proteins are small membrane-associated GTPases that play important roles as activators of developmental signaling cascades. Ras homologs are common across Eukaryota, found in organisms from fungi to mammals. In human cells, four proto-typical Ras homologs have been identified: HRas, NRas, KRas4A and KRas4B. Although these proteins demonstrate highly similar effector binding domains, they undergo different post-translational modifications (PTMs) and trafficking patterns, and thus, localize to distinct plasma membrane microdomains (Parikh et al., 2007). In the human pathogenic fungus *Aspergillus fumigatus*, two Ras proteins are produced: one with high similarity to the human HRas, designated RasA, and a second homolog, designated RasB, that is produced by filamentous fungi only (Fortwendel et al., 2004). Both proteins have unique and overlapping functions in mediating fungal growth, morphogenesis, and pathogenesis (reviewed in detail by Fortwendel, 2012, 2015). Because of its major functions in fungal growth and pathogenesis, we have studied RasA extensively and have shown RasA to primarily localize to the plasma membrane (Fortwendel et al., 2012). At the plasma membrane, RasA orchestrates the complex processes of hyphal morphogenesis, asexual reproduction, cell wall stability and virulence (Fortwendel, 2015).

Ras proteins generally exhibit a conserved domain architecture. The N-terminal region of the protein, which is generally known as the G-domain, is highly conserved and is required for direct protein-protein interactions to affect downstream signaling. The G-domain harbors switch I and switch II regions, which are responsible for altering protein conformation during the transitions between active and inactive states (Vetter and Wittinghofer, 2001). In contrast, the C-terminal portion, which is known as the hypervariable region (HVR), is required for membrane localization via a post-translational lipidation and maturation pathway (**Supplementary Figure S1**). The HVR contains two major motifs for membrane targeting and stable association: a CAAX motif (C is cysteine, A is any aliphatic residue, X can be variable amino acids) and a palmitoylation motif, composed of conserved cysteine residues. For proper protein delivery and membrane association, each of these two motifs must undergo a specific set of PTMs (Ahearn et al., 2012; Al Abdallah and Fortwendel, 2015). The first type of PTM occurs at the CAAX box immediately after Ras translation and includes multiple steps. First, prenylation occurs at the cysteine residue of the CAAX box in the cytosol. Protein prenylation involves the addition of either a 15-carbon farnesyl group (a process known as farnesylation) or 20-carbon geranylgeranyl group (a process known as geranylgeranylation) to the cysteine residue of the CAAX box via a thioether bond (Zhang and Casey, 1996; Fu and Casey, 1999). The attachment of Ras proteins to the plasma membrane is predicted to be primarily initiated by farnesylation, however, the geranylgeranylation of K- and N-Ras has been observed in human colon carcinoma DLD-1 cells after treatment with farnesyltransferase inhibitor (Whyte et al., 1997). After prenylation, Ras proteins display a higher affinity for membranes due to increased hydrophobicity and become localized to the endoplasmic reticulum (ER).

On the ER, the -AAX residues are cleaved by an enzyme known as a CAAX prenyl protease (Manolaridis et al., 2013). In *Saccharomyces cerevisiae*, two CAAX prenyl proteases have been identified: Rce1p and Ste24p. Both enzymes are integral membrane metalloproteases which exhibit multiple transmembrane domains. Rce1p (Ras Converting Enzyme 1) is the major CAAX prenyl-protease, which displays activity against nearly all prenylated CAAX proteins (reviewed by Dore and Schmidt, 2013). Ste24p is a zinc metalloprotease that displays narrower substrate specificities than Rce1p (reviewed by Barrowman and Michaelis, 2013). The proteolysis of the Ras2p CAAX motif is crucial for proper plasma membrane localization in yeast cells. For example, deletion of *RCE1* delocalizes the Ras2p protein in a diffused, cytosolic pattern. In contrast, mutants lacking *STE24* display typical Ras2p plasma membrane localization. Nevertheless, when *STE24* is deleted along with *RCE1*, Ras2p forms delocalized punctate structures that are not observed in the single *RCE1* deletion mutant suggesting possible indirect contributions of Ste24p to the localization of Ras2p (Manandhar et al., 2010). After -AAX cleavage, the prenylated cysteine is methylated by isoprenylcysteine methyltransferase (ICMT) on the ER. In *Saccharomyces cerevisiae*, the 239 residue-long Ste14p protein is the sole ICMT enzyme. Deletion of *STE14* does not affect yeast cell viability. However, loss of *STE14* mislocalizes Ras2p in a diffused pattern, similar to observations with *RCE1* deletion, and results in loss of mating ability associated with a 200-fold reduction in a-factor activity (Young et al., 2001; Manandhar et al., 2010). These initial PTM steps, focused on the CAAX box, afford Ras proteins with relatively weak binding affinity to cellular membranes. Thus, a second signal is required to stabilize membrane association and promote accumulation of Ras at the plasma membrane. In H-Ras homologues, such as *A. fumigatus* RasA, this second signal is provided by palmitoylation of conserved cysteine residues adjacent to the CAAX box (Manandhar et al., 2010; Fortwendel et al., 2012). The increased hydrophobicity provided by the added palmitoyl residues increases affinity for the plasma membrane.

Ras PTM pathways have been studied extensively in humans and yeast as they represent a rich landscape of promising anti-cancer targets. Using *in silico* comparative analysis, we have previously shown that RasA PTM pathway proteins are conserved in *A. fumigatus* (Al Abdallah and Fortwendel, 2015). We have also reported that deletion of the prenyltransferase enzyme mediating protein farnesylation, one of the initial components of the Ras PTM pathway, inhibits growth, mislocalizes RasA, and reduces virulence (Norton et al., 2017). In this study, we investigate the role of the remaining post-prenylation steps – proteolysis and carboxymethylation – in RasA localization and fungal vegetative growth. Additionally, we further analyze the interplay between post-prenylation processing steps and palmitoylation at the HVR of RasA with respect to plasma membrane localization.

## Materials and Methods

### Culturing Conditions and Growth Rate Analysis

Fungal strains were maintained on Glucose Minimal Medium (GMM) agar plates (Shimizu and Keller, 2001). Conidia were produced from mycelial cultures following 3 days of growth on GMM agar plates at 37°C, and were harvested using sterile deionized water. Variations in colony morphology were analyzed by spotting 5 μl of 5000 total conidia onto the center of 60 mm GMM agar plates and incubation for 54 h at 37°C. For quantification of fungal growth rates, nutrient rich media was employed to reduce conidiation rates and allow for prolonged culture. In brief, 10 μl of 10,000 total conidia were spotted at the center of 150 mm Yeast Peptone Dextrose (YPD) agar plates (1% yeast extract, 2% peptone, 2% glucose, and 1.5% agar). Plates were incubated at 37°C and colony diameter was measured daily for 5 days. Assessment of polarity establishment during spore germination was carried out as described previously (Fortwendel et al., 2004), with some modifications. Briefly, sterilized coverslips were submerged in liquid GMM, which was then inoculated with *A. fumigatus* conidia at a final concentration of 10^5^ conidia/ml. Coverslips were inverted onto a glass slide and analyzed by microscopy after 6 and 8 h of incubation at 37°C. A total of 100 conidia and germlings from each strain were counted. Polarity establishment was defined as the production of a germ tube. Polarity establishment percentage was calculated as number of conidia with germ tube divided by the total number of counted conidia (i.e., 100 conidia). All growth experiments were performed in triplicate. Data were analyzed and presented as the mean value ± standard deviation.

### Genetic Manipulations of *A. fumigatus*

The generation and transformation of *A. fumigatus* protoplasts was performed using a previously described protocol (Yelton et al., 1984). In general, the deletion of RasA post-prenylation processing enzymes and the expression of *gfp*-tagged *rasA* at its native locus were performed by homologous recombination using hygromycin, phleomycin, or uridine/uracil auxotrophy (*pyrG*) selection cassettes flanked by 1.5 kb arms upstream and downstream of the corresponding target gene. The control Δ*akuB*-*pyrG*^+^ strain was constructed by replacing the non-functional *pyrG* locus of the *A. fumigatus* KU80ΔpyrG strain (da Ferreira et al., 2006) with the functional *pyrG* homologue from *A. parasiticus*. Similarly, the generation of a single deletion mutant of *cppB* (*RCE1* homolog) and *cppA* (*STE24* homolog) genes was performed using the *A. parasiticus pyrG* auxotrophic marker in the KU80ΔpyrG strain. The Δ*cppA* mutant was then used as a recipient strain for the generation of a *cppB*/*cppA* double-deletion mutant using a hygromycin resistance cassette. To construct a partial complementation strain, where the *cppB* coding sequence was restored in the Δ*cppB*/Δ*cppA* mutant, the *cppB* genomic locus, including 1.8 kb upstream and 0.4 kb downstream of the predicted coding sequence, was PCR amplified and fused to a phleomycin resistance cassette using an overlap extension PCR method. The resulting construct was ectopically integrated into the Δ*cppB*/Δ*cppA* strain and single copy integration was confirmed by Southern blot. Deletion of the *icmA* (*STE14* homologue) gene was carried out using a hygromycin resistance cassette in the Δ*akuB*-*pyrG*^+^ control strain. For the expression of *gfp*-tagged wild type *rasA*, and the palmitoylation mutants *rasA^C206S^* and *rasA^C2067^* in *A. fumigatus*, GFP was fused to the N-terminus of *rasA, rasA^C206S^* and *rasA^C207S^* by standard cloning techniques using a vector containing eGFP and a phleomycin resistance cassette.

### Analysis of Response to Cell Wall-Active Compounds

Single- and double deletion mutants of the RasA post-prenylation modifying enzymes were tested for their sensitivity to the cell wall stressing agents, Calcofluor White (CFW) and Congo Red (CR), using a modified protocol of a previously described spot inoculation assay (Mrša and Tanner, 1999; Ram and Klis, 2006). Briefly, 10-fold serial dilutions of conidia from each strain were prepared and 10 μl of each dilution was spotted on GMM agar plates containing 150 μg/ml CFW or 40 μg/ml CR. Plates were incubated at 37°C for 2 days (for CFW) and 3 days (for CR). Strains were assessed for sensitivity to CFW and CR by analyzing colony growth over the inoculum dilution range. *In vitro* susceptibility of *A. fumigatus* strains to Caspofungin and Nikkomycin Z was performed using a modified protocol of broth microdilution method (Pfaller et al., 2000, 2003). Briefly, Caspofungin (10 mg/ml) and Nikkomycin Z (10 mg/ml) stocks were prepared in water and stored at -20°C until use. A dilution range of each drug from 0.1 to 100 μg/ml were tested against conidia at a final inoculum of 5 × 10^4^/ml in GMM. Assay plates were incubated at 37°C for 24–48 h. For visualization of Caspofungin-induced morphological changes by microscopy, sterilized coverslips were submerged in GMM broth supplemented with Caspofungin (25 μg/ml). Next, conidia of each strain were used to inoculate the GMM broth at a final concentration of 5 × 10^4^ conidia/ml. Following 24 h incubation at 37°C, each strain was visualized under the microscope and the morphological changes of the mutants were recorded. Lysed germlings were defined as germinating conidia that had produced germ tubes but displayed visible disruption of the germ tube with overt cytoplasmic leakage.

### Ras Activation Assay

The Ras activation assay was performed according to a previously described protocol for the Ras Activation Assay Kit (EMD Millipore) after optimization for *A. fumigatus* RasA (Al Abdallah et al., 2016). Briefly, conidia were inoculated in liquid GMM at a final concentration of 1 × 10^6^ conidia/ml and incubated overnight at 37°C and 250 rpm. Mycelia were flash frozen in liquid nitrogen and homogenized using a mortar and pestle. The crushed mycelia were resuspended in 1:1 v/v ratio of lysis buffer (25 mM Tris-HCl [pH 7.5], 20 mM MgCl_2_, 75 mM NaCl, 1 mM EDTA, 1% Igepal CA-630, 2% glycerol, 1:100 Pefabloc, 1:100 protein inhibitor cocktail). The insoluble cell wall debris were removed by centrifugation at 3500 rpm, 4°C, for 8 min. The supernatant was transferred to a new tube and analyzed for total protein concentration via Bradford assay. For the isolation of active RasA, 5 mg of total protein was incubated with Raf1-RBD coated beads for 45 min at 4°C. RasA-bound beads were centrifuged at 13,000 rpm for 10 s. The pellet was washed 3 times in 0.5 ml lysis buffer, resuspended in 40 μl of lysis buffer and boiled for 10 min. As a control for total RasA levels, 50 μg of total protein was similarly boiled for 10 min. Both, samples were then separated on a 12% SDS-polyacrylamide gel (Bio-Rad), transferred to a polyvinylidene difluoride (PVDF) membrane and probed with “THE^TM^ GFP,” a rabbit monoclonal antibody against GFP (diluted 1:2500, GenScript, Cat. # A01704), followed by a horseradish peroxidase (HRP)-conjugated goat anti-rabbit IgG (diluted 1:5000, Santa Cruz, Cat. # SC2004). Membranes were imaged using a Bio-Rad ChemiDoc XRS HQ System and QuantityOne software (v4.6.5, Bio-Rad). The densitometric analysis of active RasA was computed and normalized to total RasA in each strain by using ImageJ software (Schneider et al., 2012). The assays were performed in triplicate. Data were analyzed and presented as the mean value ± standard deviation.

### Fluorescent Microscopy

For the analysis of subcellular localization of wild type and mutated GFP-tagged RasA, *A. fumigatus* conidia (final concentration 1 × 10^5^/ml) were incubated over sterile coverslips submerged in liquid GMM at 37°C for the indicated durations. Coverslips were then washed with deionized water and fluorescence microscopy was performed on different stages of vegetative growth using a Nikon NiU microscope equipped with GFP filter settings.

## Results

### Deletion of the CAAX Prenyl-protease, *cppB*, Slows Hyphal Growth and Delays Conidial Germination

Previously, we reported the identification of putative genes that display high similarity to the known yeast post-prenylation processing enzymes (Al Abdallah and Fortwendel, 2015). Based on their predicted functions, these enzymes were designated CppB (Afu6g04890) and CppA (Afu4g07590) for **C**AAX **P**renyl **P**rotease Family 2 and Family 1, and IcmA (Afu2g08420) for **I**soprenylcysteine **C**arboxyl **M**ethyltransferase. These enzymes are homologues of *S. cerevisiae* Rce1p, Ste24p, and Ste14p, respectively. To determine the significance of the post-prenylation pathway for growth and development in *A. fumigatus*, the genes that encode CppB, CppA, and IcmA were deleted. To study the effect of complete elimination of CAAX prenyl-protease activity, a double deletion mutant of *cppB* and *cppA* was also generated. Because methylation of proteins processed by this pathway is directly dependent on proteolysis of the CAAX box, this double deletion mutant is expected to block further processing of protein substrates by IcmA. To construct a reliable strain for growth comparisons, the uracil-auxotrophic Δ*akuB*^ΔKU80^ parent strain was also complemented to prototrophy. This new control strain, designated Δ*akuB*-*pyrG*^+^, was generated by integrating the *A. parasiticus pyrG* gene into the non-functional *A. fumigatus* Δ*akuB*^ΔKU80^ *pyrG* locus. The Δ*akuB*-*pyrG*^+^ strain was employed as a growth control strain for the work described herein.

**FIGURE 1:**
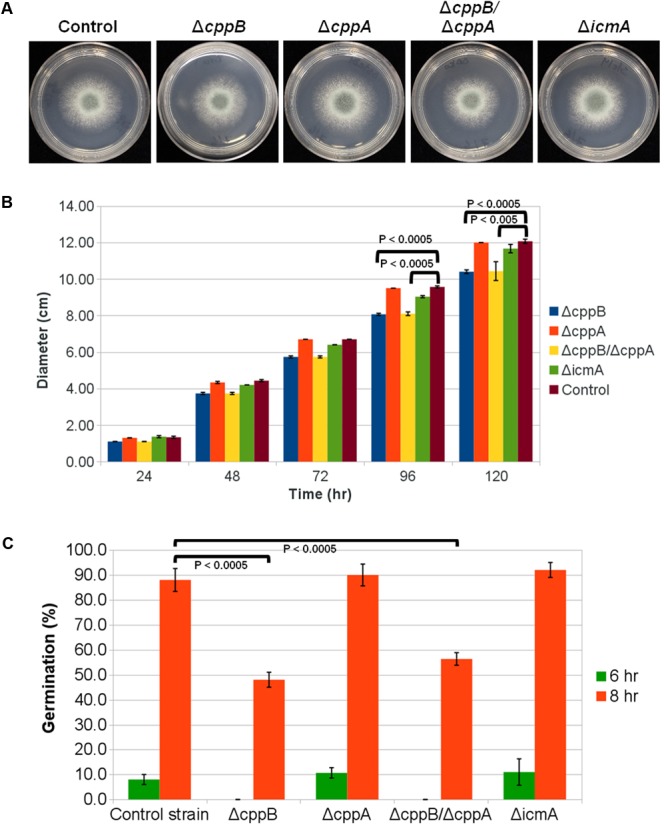
Deletion of the *RCE1*-homolog *cppB* in *A. fumigatus* reduces hyphal growth and delays establishment of polarity. **(A)** Colony morphology of mutants lacking RasA post-prenylation modifying enzymes in comparison to the control strain Δ*akuB-pyrG^+^*. For each strain, 5000 conidia were spotted onto the middle of GMM agar plates and allowed to grow for 2 days at 37°C. **(B)** Quantification of colony diameter of the strains deficient in post-prenylation processing enzymes in comparison to the control strain Δ*akuB-pyrG^+^*. Conidia (10^4^) of each strain were spotted on the middle of YPD agar plates. The plates were incubated at 37°C and the colony diameter was measured daily for 5 days. Measurements represent the average diameter of three independent experiments for each strain at the indicated timepoint. Error bars represent the standard deviation of the three independent experiments for each strain at the indicated timepoint. **(C)** Conidial germination rates of the deletion mutants of the post-prenylation processing pathway. GMM broth was inoculated with conidia of each strain at a final concentration of 10^5^ conidia/ml and incubated at 37°C until the indicated timepoint. Polarity establishment was presented as the percentage of conidia forming a germtube from the total number of counted conidia for each strain at the indicated timepoint. Measurements and error bars represent the average and standard deviation of 3 independent experiments. Statistical comparisons were completed using the Student’s *T*-test two-sample assuming equal variances. The colony diameter quantification and conidial germination rates for the complement strain Δ*cppB*/Δ*cppA+cppB* are presented in **Supplementary Figure S2**.

To delineate roles for the post-prenylation pathway in *A. fumigatus* development, colony morphology and growth rates were observed for each mutant and compared to the control strain. Deletion of *cppB, cppA* or *icmA* alone or double deletion of *cppB* and *cppA* did not alter colony morphology or impair hyphal morphogenesis (**Figure 1A** and data not shown). However, the Δ*cppB* and Δ*cppB*/Δ*cppA* mutants displayed slightly slower growth rates than the control strain as the 5-day colony diameter of both mutants was reduced by approximately 17% (**Figure 1B**). Although double deletion of both CAAX prenyl proteases reduced hyphal growth, deletion of *cppA* alone did not affect fungal growth (**Figure 1B**). Additionally, reconstitution of the *cppB* gene in the Δ*cppB*/Δ*cppA* double mutant restored growth to control strain levels (**Supplementary Figure S2**). These data suggest that only *cppB* plays a role in *A. fumigatus* hyphal growth.

Previously, we reported that deletion of *ramA*, the *A. fumigatus* farnesyltransferase β-subunit, causes a reduction in the viability of conidia as well as a delay in germination rate (Norton et al., 2017). To analyze the effects of the post-prenylation processing steps on the early stages of *A. fumigatus* polarity establishment, we measured the germination rates of the *cppB, cppA, icmA* and *cppB*/*cppA* deletion mutants after 6- and 8-h of culture in minimal media. Similar to the growth rate data, conidia from the Δ*cppB* and Δ*cppB*/Δ*cppA* mutants exhibited delayed germination. After 6 h, no germ tubes were observed in these two mutants. In contrast, 8% of the conidia from the control strain had formed a germ tube at this timepoint. Approximately 50% of conidia from the Δ*cppB* and Δ*cppB*/Δ*cppA* mutants germinated after 8 h versus 88% of the conidia from the control strain. Conidia from the Δ*cppA* and Δ*icmA* mutants displayed germination levels similar to that of the control strain after 6 and 8 h of growth (**Figure 1C**). Again, complementation of the *cppB* gene in the Δ*cppB*/Δ*cppA* double mutant restored germination rates to levels comparable to the control strain (**Supplementary Figure S2**). Taken together, these data suggest that post-prenylation processing does not play a major role in fungal growth and development.

### Loss of *cppB* Results in Hypersensitivity to Cell Wall-Active Agents

**FIGURE 2:**
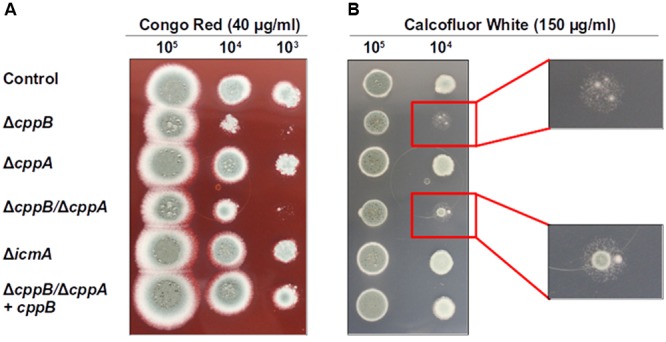
Deletion of *cppB* increases cell wall sensitivity to **(A)** Congo red (CR) and **(B)** Calcofluor white (CFW). Strains were spotted in 10-fold dilutions onto GMM agar plates supplemented with CR (final concentration 40 μg/ml) and CFW (final concentration 150 μg/ml) and grown at 37°C for 66 h (for CR) and 48 h (for CFW). The control strain Δ*akuB-pyrG^+^* and the complement strain Δ*cppB*/Δ*cppA+cppB* were used for comparison. Enlargements for **(B)** detail the similar phenotypes of strains lacking *cppB* under CFW stress.

Ste24p, one of the yeast CAAX prenyl proteases, is known to play a role in chitin biosynthesis in yeast, although the exact mechanism remains unclear (Meissner et al., 2010). To investigate the role of post-prenylation CAAX box processing in cell wall stability in *A. fumigatus*, we first examined growth inhibition in response to the cell wall perturbing agents Calcofluor White (CFW) and Congo Red (CR). CFW and CR preferentially bind chitin and β-1,3 glucans, respectively, in the fungal cell wall to cause toxic effects in fungi (Roncero and Durán, 1985; Hill et al., 2006; Ram and Klis, 2006). Sensitivity to CFW and CR was performed by a spot inoculation assay employing the Δ*cppB*, Δ*cppA*, Δ*icmA* and Δ*cppB*/Δ*cppA* mutants. Loss of the *RCE1* homologue, *cppB*, resulted in hypersensitivity to both cell wall stress agents and the deletion of both CAAX protease enzymes (Δ*cppB*/Δ*cppA*) showed a similar increase in sensitivity (**Figures 2A,B**). Culture in the presence of CFW resulted primarily in lack of colony growth when coupled with loss of *cppB*, however, both the Δ*cppB* and Δc*ppB*/Δ*cppA* mutants developed slow-growing microcolonies under these conditions (**Figure 2B**). Reconstitution of *cppB* in the Δ*cppB*/Δ*cppA* mutant resulted in a return of cell wall stress tolerance to levels comparable to the control strain (**Figures 2A,B**). In addition, the loss of *cppA* alone, or the loss of *icmA*, resulted in no changes to cell wall stress tolerance (**Figures 2A,B**). Together, these results suggested that *cppB* alone contributes to cell wall stability in *A. fumigatus*.

**FIGURE 3:**
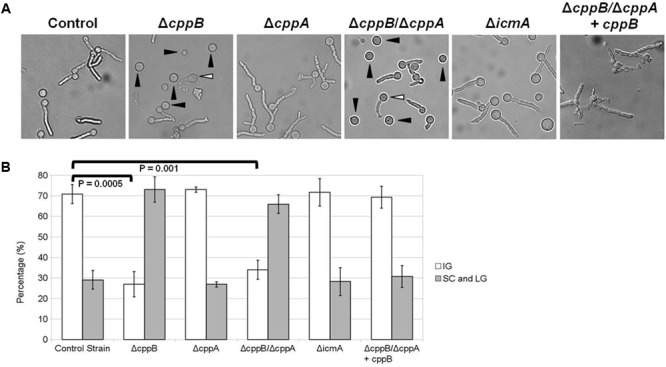
Deletion of *cppB* causes hypersensitivity to Caspofungin. **(A)** Morphological changes of the mutants in response to Caspofungin (25 μg/ml). Conidia of each strain were used to inoculate GMM broth at final concentration of 5 × 10^4^/ml and incubated at 37°C for 24 h. **(B)** Quantification of the mutant hypersensitivity to Caspofungin. Measurements and error bars represent the average and standard deviation of 3 independent experiments. The control strain Δ*akuB-pyrG^+^* and the complement strain Δ*cppB*/Δ*cppA+cppB* were used for comparison. IG: Intact Germlings, SC: Swollen Conidia (denoted by black arrowheads in the Δ*cppB* and Δ*cppB*/Δ*cppA* panels), LG, Lysed Germlings (denoted by white arrowheads in the Δ*cppB* and Δ*cppB*/Δ*cppA* panels). Statistical analyses were performed using the Student’s *T*-test two sample assuming equal variances and denote the difference in intact germlings formed by the control strain, Δ*cppB* and Δ*cppB*/Δ*cppA* mutants.

To further investigate whether the *cppB* deletion mutant displays a sensitivity to specific inhibition of either of the two major cell wall components, β-glucan or chitin, the mutant strains were exposed to Caspofungin or Nikkomycin Z. Caspofungin is a member of the echinocandin antifungals that specifically targets β-1,3-D-glucan synthase, and thus disrupts cell wall stability (Bowman et al., 2002; Saravolatz et al., 2003). In contrast, Nikkomycin Z is a specific inhibitor of chitin synthases and disrupts cell wall stability via downregulation of chitin synthesis (Ganesan et al., 2004). Using broth microdilution assay, no change in susceptibility patterns was observed for the mutant strains within a range of Caspofungin concentrations (0.25 – 1 μg/ml) that are inhibitory for wild type *A. fumigatus* (data not shown). Inhibition of *A. fumigatus* growth by Caspofungin treatment is often characterized by a paradoxical effect, where higher concentrations of Caspofungin are less growth inhibitory than lower concentrations (Lamoth et al., 2014). No change in the paradoxical effect of Caspofungin treatment was observed for any of the mutant strains (data not shown). At a higher concentration of Caspofungin, deficiencies in the establishment and maintenance of hyphal growth became apparent for the Δ*cppB* mutant. After 24 h of growth in the presence of Caspofungin (25 μg/ml), 71% of conidia from the control strain had developed into intact germlings (IG) as defined by the presence of non-lysed, slow-growing germ tubes (**Figures 3A,B**). These results were similar for both the Δ*cppA* and Δ*icmA* mutants (**Figures 3A,B**). The remaining conidia (∼29%) from the Δ*cppA*, Δi*cmA* and control strains developed into germlings that displayed overt cell lysis (lysed germlings (LG)) or remained as swollen conidia (SC) that failed to establish polarity (**Figures 3A,B**). In contrast, microscopic examination of the Δ*cppB* and Δ*cppB*/Δ*cppA* mutants revealed a significant reduction in the number of IG (∼27% and ∼34%, respectively) and increased number of SC/LG (∼73% and ∼66%, respectively) (**Figures 3A,B**). Restoration of *cppB* in the Δ*cppB*/Δ*cppA* mutant restored tolerance to high concentrations of Caspofungin (**Figures 3A,B**). Using a broth microdilution assay, we next examined sensitivity of the mutant strains to treatment with Nikkomycin Z. In contrast to Caspofungin, no differences in growth inhibition among the mutant and control strains were noted across a broad range of Nikkomycin Z concentrations (0.1 – 100 μg/ml) (data not shown). Taken together, these data suggest that CppB modulates cell wall biosynthesis and integrity, and specifically impacts pathways protecting germlings from lysis in response to high concentrations of Caspofungin.

### CAAX Proteolysis Is Not Required for RasA Plasma Membrane Localization or Activation

**FIGURE 4:**
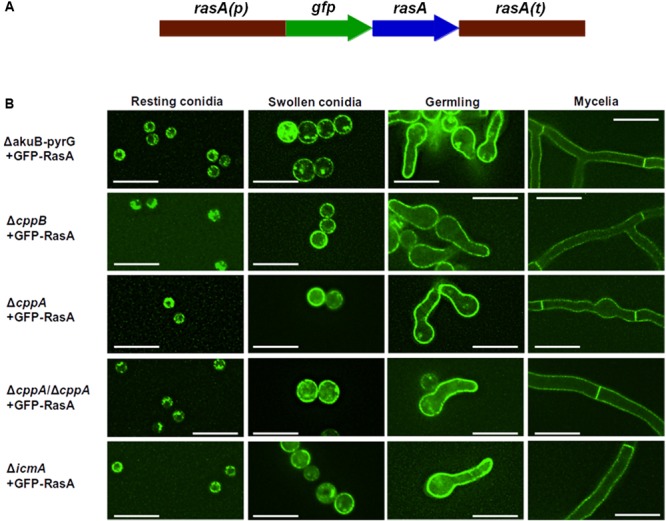
The post-prenylation processing pathway is dispensable for RasA localization to the plasma membrane. **(A)** Schematic overview of *gfp*-*rasA* gene fusion. Full-length *rasA* was fused in-frame to *gfp* under the control of native *rasA* promoter and 3′ UTR. **(B)** Localization of RasA was observed during different stages of fungal growth (indicated at the top of each column). The control strain Δ*akuB-pyrG^+^* was used for comparison. Scale bar = 10 μM.

**FIGURE 5:**
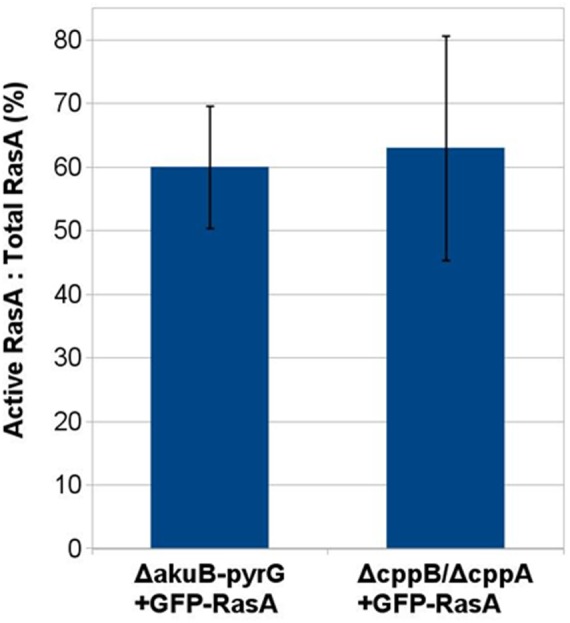
Post-prenylation processing does not impact RasA activation. Densitometric analysis of a immunoblot-based Ras activity assay was carried out using ImageJ software. No statistically significant difference was noted between the GFP-RasA-expressing Δ*akuB-pyrG^+^* control strain and the Δ*cppB*/Δ*cppA* mutant. Measurements and error bars represent the average and standard deviation of three independent experiments.

RasA plasma membrane localization is essential for biological processes such as polarized growth, cell wall integrity, and virulence in *A. fumigatus* (Fortwendel et al., 2012). Farnesylation and subsequent palmitoylation, have both been shown to be critical for the proper localization of RasA to the plasma membrane (Fortwendel et al., 2012; Norton et al., 2017). To inspect the role of the post-prenylation pathway in RasA localization, a GFP-tagged RasA fusion protein was expressed under the RasA native promoter in the Δ*cppB*, Δ*cppA*, Δ*icmA* and Δ*cppB*/Δ*cppA* mutants (**Figure 4A**). To examine if the post-prenylation pathway regulates RasA localization throughout *A. fumigatus* development, conidia from each strain were cultured to identical phases of growth and analyzed by fluorescent microscopy. To account for the slight delay in germination caused by *cppB* deletion, all strains were cultured to the same growth phase rather than to a specific timepoint. These growth phases included: (1) resting conidia, (2) swollen conidia, (3) germlings, and (4) mycelia. Localization of the GFP-RasA signal was similar in all strains at each growth phase. Resting conidia displayed a mixture of cytoplasmic, punctate and plasma membrane localization whereas swollen conidia for each strain exhibited a clearer localization of GFP-Ras to the cell periphery (**Figure 4B**). Both germling and mature mycelia of all strains displayed strong plasma membrane association (**Figure 4B**). These findings suggest that the post-prenylation processing pathway does not play a major role in the accumulation of RasA at the *A. fumigatus* plasma membrane during growth. To study the effect of CAAX proteolysis on RasA activity levels, a Ras activation assay was also employed. Using this assay, GTP-bound Ras was precipitated with agarose beads coated with a Ras-binding domain (RBD) peptide isolated from the Ras effector kinase Raf-1 (Al Abdallah et al., 2016). The ratio of active Ras to total Ras was then quantified by western blot analysis using an anti-GFP antibody to detect precipitated Ras proteins. The Δ*cppB*/Δ*cppA+*GFP-RasA strain, generated for the RasA localization studies, was used for this study as this strain completely lacks CAAX proteolysis activity, blocking downstream processing by IcmA, and displays growth reductions similar to that of the *cppB* single deletion mutant. The Δ*akuB*-*pyrG*+GFP-RasA strain, which was also generated for the RasA localization studies, was used as a control. Using protein lysates extracted from mature mycelia, no significant changes in RasA activation levels were observed in the Δ*cppB*/Δ*cppA* background when compared to the control strain (**Figure 5**). Taken together, these data suggest that the post-prenylation pathway is dispensable for appropriate localization and activation of RasA.

### Palmitoylation and CAAX Proteolysis Cooperate to Ensure Plasma Membrane Presence of RasA During Hyphal Growth

**FIGURE 6:**
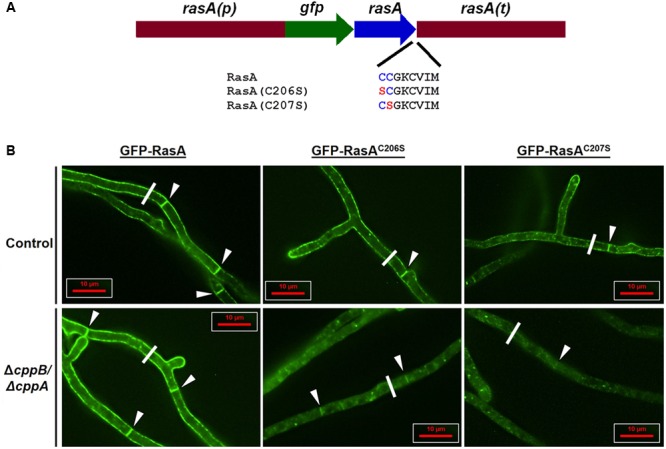
RasA palmitoylation and CAAX proteolysis cooperate for RasA plasma membrane localization. **(A)** Schematic overview of RasA dual palmitoylation motif mutants RasA^C206S^ and RasA^C207S^ fused in-frame to GFP. The wild type construct *gfp*-*rasA*, and the dual palmitoylation mutants *gfp*-*rasA^C206S^* and *gfp*-*rasA^C207S^* were expressed in the control strain Δ*akuB-pyrG^+^* and the Δ*cppB*/Δ*cppA* mutant under the control of *rasA* promoter and 3′ UTR. **(B)** Subcellular localization of RasA, RasA^C206S^, and RasA^C207S^ in the control strain Δ*akuB-pyrG^+^* and the Δ*cppB*/Δ*cppA* mutant was observed in growing hyphae. Significant accumulation of RasA in the cytoplasm and on internal structures was observed when palmitoylaytion deficiency (GFP-RasA^C206S^ or GFP-RasA^C207S^) was coupled with loss of CAAX proteolysis (Δ*cppB*/Δ*cppA*). The white line shown in each panel represents the cross-section employed for pixel intensity analysis to quantify RasA distribution in each strain. The pixel intensity graph produced by data acquired from the micrographs shown in **(B)** are provided as “Replicate 1” in **Supplementary Figure S3**. Pixel intensity graphs for two additional replicates from each strain, taken from randomly chosen hyphal segments, are also shown in **Supplementary Figure S3.**

The localization of Ras proteins to the plasma membrane is mediated by two motifs that are located at the HVR. In contrast to our findings reported herein and results previously published with *Cryptococcus neoformans* (Esher et al., 2016), deletion of the CAAX protease-coding *RCE1* gene mislocalizes Ras2p from the plasma membrane in *S. cerevisiae*. Therefore, we predicted that variations within the HVR of RasA and Ras2p may be responsible for the differential dependence of Ras protein localization on CAAX proteolysis between *A. fumigatus* and *S. cerevisiae*. In addition to the CAAX box, one of the major motifs that drives Ras localization to the plasma membrane is the palmitoylation motif. In the RasA protein of *A. fumigatus*, this motif is composed of a “dual-cysteine” region at residues 206 and 207 (Fortwendel, 2015). Mutation of both cysteines completely mislocalizes RasA to internal membranes and leads to severe hyphal abnormalities in *A. fumigatus* (Fortwendel et al., 2012). In contrast, the palmitoylation motif in Ras2p of *S. cerevisiae* contains only a single palmitoylation site (Fortwendel, 2015). Because membrane affinity is increased by the addition of hydrophobic palmitoyl moieties, we hypothesized that a weaker membrane binding affinity caused by uncleaved -AAX residues in the double mutant (Δ*cppB*/Δ*cppA*) may be overridden by a strong signal from the “dual-palmitoylation” motif in *A. fumigatus*. To test this hypothesis, we studied the interplay between cysteine palmitoylation and -AAX proteolysis of the RasA HVR. A series of single cysteine-to-serine mutations of the palmitoylation motif residues 206 and 207, which we refer to here as RasA^C206S^ and RasA^C207S^, were generated by PCR. The resulting RasA mutant alleles were fused to GFP under the expression of the endogenous *rasA* promoter (**Figure 6A**). Our previous studies have shown that expression of Ras alleles that are mutated at the palmitoylation motif or fused with GFP at the N-terminus does not negatively impact RasA protein levels in total lysates (Fortwendel et al., 2012). The constructs, GFP-RasA^C206S^ and GFP-RasA^C207S^, were then targeted for integration at the native *rasA* genomic locus in the Δ*cppB*/Δ*cppA* double mutant and the Δ*akuB*-*pyrG*^+^ control strain. Although our assays detected no phenotypic changes in the Δ*cppA* mutant, implying more important roles for *cppB*, it is possible that even inefficient CAAX proteolysis via CppA could contribute to RasA localization. Therefore, we utilized the Δ*cppB*/Δ*cppA* mutant for our localization studies to ensure all cellular CAAX proteolysis activity is absent.

As detected by deconvolution fluorescent microscopy, mutation of either cysteine residue resulted in a moderately increased accumulation of GFP signal on internal structures in the control strain with the C207 residue playing a more prominent role in RasA plasma membrane localization (**Figure 6B**). These data were in agreement with earlier reports of the localization of palmitoylation-deficient RasA mutants in a different genetic background of *A. fumigatus* (Fortwendel et al., 2012). In contrast, significant accumulation of RasA in the cytoplasm and on internal structures was noted for the Δ*cppB*/Δ*cppA* double mutant when either GFP-RasA^C206S^ and GFP-RasA^C207S^ was expressed (**Figure 6B**). Although significant mislocalization was noted for both cysteine residue mutants in Δ*cppB*/Δ*cppA*, the GFP-RasA^C207S^ fusion protein appeared mislocalized to an even greater extent than GFP-RasA^C206^. This was evident by the extremely weakly fluorescent septa in the Δ*cppB*/Δ*cppA* mutant expressing GFP-RasA^C207S^ (**Figure 6B**). As expected, localization of RasA proteins with intact palmitoylation motifs in both the control strain Δ*akuB*-*pyrG*^+^ and the Δ*cppB*/Δ*cppA* mutant displayed strong plasma membrane presence (**Figure 6B**). Multiple cross-sections of hyphae from each strain were analyzed for variations in pixel intensity to quantitate GFP-RasA distribution across the hyphal diameter. The results confirmed that loss of CAAX proteolysis exacerbated RasA mislocalization induced by palmitoylation deficiency (**Supplementary Figure S3**). Surprisingly, although localization of RasA was significantly affected when CAAX proteolysis and palmitoylation were inhibited in tandem, neither mutation resulted in alteration of colony morphology or growth rate deficiencies when compared to controls (**Supplementary Figure S4**). These findings suggest that even minimal accumulation of RasA at the plasma membrane is sufficient to support wild type hyphal growth and morphogenesis in *A. fumigatus*. These data also suggest that dual-palmitoylation events occurring within the Ras HVR in fungi like *A. fumigatus* and *C. neoformans* may provide enough stable membrane association to override negative effects from inhibition of CAAX proteolysis.

## Discussion

Although the Ras post-prenylation pathway has been characterized in mammalian cells as well as the budding-yeast *S. cerevisiae* and the yeast-like fungus *C. neoformans*, this is the first study to characterize Ras post-prenylation modifying enzymes in a filamentous fungus. Additionally, this study is the first investigation of the direct interplay between post-translational modification steps of the RasA CAAX box and the palmitoylation cysteine motif in fungi. The significance of post-prenylation processing enzymes varies among eukaryotes. For example, in the mammalian cell, *Rce1* (*cppB* homologue)- and *Icmt* (*icmA* homolog)-deficient fibroblasts display slower growth rates than wild type cells, however, the effects of *Icmt* deletion in mice are more severe than *Rce1* deficiency. Additionally, Ras proteins are significantly mislocalized from the plasma membrane in *Rce1*- and *Icmt*-deficient fibroblasts (Kim et al., 1999; Bergo et al., 2002, 2004). Similarly, in *S. cerevisiae*, mislocalization of Ras1p and Ras2p from the plasma membrane has been reported in *RCE1*- and *STE14*-deletion mutants (Manandhar et al., 2010). In the human pathogenic fungus *C. neoformans*, deletion of the *RCE1* or *STE14* genes does not affect Ras1 membrane localization, but nevertheless reduces fungal growth and slightly impairs virulence (Esher et al., 2016). Although Ras localization and activation are regulated by two distinct domains, the majority of Ras signaling functions, including the orchestration of germination and hyphal morphogenesis, are dependent on its proper localization to the plasma membrane. Therefore, we first aimed to examine the effect of *cppB, cppA* and *icmA* deletion on germination, morphogenesis and RasA localization and subsequent activation.

We began our analysis by deleting the RasA post-prenylation pathway genes (i.e., *cppB, cppA* and *icmA*), including a double-deletion of the two CAAX proteases (Δ*cppB*/Δ*cppA*). Interestingly, although the number of predicted and known proteins containing a CAAX motif is quite large in *A. fumigatus (*Norton et al., 2017*)*, the post-prenylation processing pathway mutants did not exhibit severe growth abnormalities. This initial finding indicates that CAAX post-prenylation processing of this large number of proteins in *A. fumigatus* may not be crucial for their function. To more closely examine growth aberrancies, we also characterized the roles of the post-prenylation modifying enzymes in conidial germination. Germination of *A. fumigatus* conidia is mediated by Ras, Ras-like, and Rho proteins (Boyce and Andrianopoulos, 2006; Fortwendel et al., 2011; Park and Yu, 2016). Therefore, improper processing of the Ras CAAX motif could be predicted to alter this cellular process. For example, we have previously shown that farnesylation of the *A. fumigatus* RasA CAAX motif is required for conidial viability and germination rate (Norton et al., 2017). In contrast, our data clearly show that only deletion of *cppB* delayed conidial germination but did not affect viability, whereas deletion of the other RasA post-prenylation pathway genes did not affect conidial germination or viability. Furthermore, we examined the importance of post-prenylation processing on RasA plasma membrane localization. While prenylation and the post-prenylation modifications are required for proper localization of Ras in yeast (Manandhar et al., 2010) and mammalian cells (Michaelson et al., 2005), only farnesyltransferase deletion impairs Ras localization to the plasma membrane in the human pathogenic fungus *C. neoformans* (Esher et al., 2016). In *A. fumigatus*, we have previously shown that the post-translational modifications of RasA at the CAAX motif are necessary for the localization of RasA to the plasma membrane. For example, mutagenesis of the CAAX cysteine residue (C210) completely mislocalizes RasA to the cytoplasm and results in a non-functional Ras protein (Norton and Fortwendel, 2014). Additionally, deletion of the farnesyltransferase β-subunit, *ramA*, partially mislocalizes RasA to the cytoplasm (Norton et al., 2017). When we investigated the effect of post-prenylation pathway in this work, we did not observe any significant change in RasA plasma membrane localization suggesting negligible effects of post-prenylation pathway on RasA localization. Taken together, we conclude that the post-prenylation modifying enzymes play only minor roles in *A. fumigatus* vegetative growth and RasA localization.

Although major defects in growth and germination were not found, we did note a decrease in tolerance to cell wall stress. When we tested the mutants for cell wall integrity using Congo Red (CR), Calcofluor white (CFW), and Caspofungin, both the Δ*cppB* and Δ*cppB/*Δ*cppA* mutants displayed higher sensitivity in comparison to the control strain toward all tested stressing agents. Our data are consistent with previous work in *S. cerevisiae*, where deletion of *RCE1* led to a significant increase in cell susceptibility to CFW (Meissner et al., 2010). In contrast, the Δ*STE24* mutant exhibited higher levels of resistance toward CFW (Meissner et al., 2010). This is distinct from our data, which showed that deletion of *cppA* (*STE24* homolog) did not alter fungal susceptibility to CFW. Although it is unclear why the Δ*cppB* and Δ*cppB/*Δ*cppA* mutants exhibited higher sensitivity levels toward cell wall-active compounds, these phenotypes are likely driven by dysregulation of cell wall stress and biosynthetic networks. Several proteins known or predicted to regulate cell wall stability and biosynthesis contain CAAX motifs and their incomplete maturation through loss of the post-translational modifying enzyme studied herein could cause minor aberrancies in stress tolerance. These proteins, at a minimum, include RasA, RhoA (Rho1), Rho2, Rho3, Rho4 and two homologs of a *S. cerevisiae* chitin synthase regulatory protein (Norton et al., 2017). However, the finding that the Δ*cppB* and Δ*cppB/*Δ*cppA* mutants exhibited sensitivity levels comparable to the control strain when treated with Nikkomycin Z suggests that the observed increase in sensitivity of these mutants may not be underpinned by decreased chitin synthase activity. The exact mechanism(s) through which the post-prenylation PTM pathway regulate cell wall stress tolerance is under further investigation.

Because CAAX proteolysis in *A. fumigatus* and *S. cerevisiae* appears to play distinct roles in Ras protein subcellular localization, we sought to explore molecular mechanisms that might underlie these differences. One of the major differences between these two proteins is the presence of a dual-cysteine palmitoylation motif within the *A. fumigatus* RasA HVR. In contrast, *S. cerevisiae* Ras2p has only a single, conserved cysteine palmitoylation motif (Nichols et al., 2009). Both cysteines of the RasA palmitoylation motif are essential for stable association of RasA with the plasma membrane (Fortwendel et al., 2012). Therefore, we hypothesized that the dual-cysteine palmitoylation motif of *A. fumigatus* RasA could provide a stronger plasma membrane association in comparison with the single-cysteine palmitoylation motif of yeast Ras2p. Our data suggest that weakened membrane binding affinity caused by an uncleaved CAAX motif in the *cppB*/*cppA* double deletion mutant can be overridden by the dual-palmitoyl groups at the RasA HVR. A synergistic interaction between multiple lipid PTMs on the proper localization of Ras proteins has been previously documented. For example, in human H- and N-Ras, farnesylation is not solely sufficient for establishing a stable association withthe plasma membrane, as the Ras-farnesyl protein dissociates from the plasma membrane within a few minutes (Resh, 2006; Aicart-Ramos et al., 2011). Therefore, a second signal is required to enhance plasma membrane association. This second signal consists of a palmitoyl moiety, conferring increased hydrophobicity to the C-terminus. The presence of a second palmitoyl lipid moiety increases the stability of Ras-plasma membrane association by several fold (reviewed by Resh, 2006).

Taken together, our findings support the hypothesis that the dual-palmitoylation motif, found mainly in RasA homologs from fungal organisms that undergo prolonged filamentous growth, may function to ensure sustained plasma membrane presence of RasA in support to hyphal morphogenesis and elongation. However, the data provided here also suggest that proper post-prenylation processing of the entire complement of CAAX motif proteins is not essential to the normal growth of *A. fumigatus* and that CAAX proteolysis through CppB appears to play a minor role in cell wall stress resistance. Future studies will focus on delineating the CppB substrate proteins that are essential to this cell stress resistance and uncovering how CAAX proteolysis modulates their specific activities.

## Author Contributions

QAA performed the studies reported herein, analyzed the data, and wrote the majority of the manuscript. AM-V, ACOS, and WG aided in the interpretation and presentation of the results reported in this manuscript. JF conceived of the majority of this work, aided in manuscript preparation, and acquired funding to support the studies described.

## Conflict of Interest Statement

The authors declare that the research was conducted in the absence of any commercial or financial relationships that could be construed as a potential conflict of interest.

## Supplementary Material

FIGURE S1 The post-prenylation CAAX maturation pathway. Shown is the previously characterized post-prenylation CAAX maturation pathway in *Saccharomyces cerevisiae*, with the RasA homolog, Ras2, as the substrate protein. Nascent Ras2 is a cytoplasmic protein that is prenylated by a prenyltransferase complex, typically farnesyltransferase, that is represented by it’s alpha-subunit, Ram1. After prenylation, Ras2 becomes associated with the endoplasmic reticulum (ER) membrane where the CAAX motif is proteolyzed by Rce1 (removing the –AAX residues) and subsequently methylated by the isoprenylcysteine-methyltransferase, Ste14. As a final step before trafficking to the plasma membrane, Ras2 is palmitoylated by a palmitoyltransferase complex (represented by Erf2) on a conserved cysteine residue upstream of the CAAX motif. This palmitoylation event provides greater hydrophobicity to the protein and drives Ras2 to the plasma membrane. The *A. fumigatus* homologs for each post-prenylation CAAX maturation component are shown below the figure. For Rce1, two potential homologs exist and have been named CppB and CppA. Although both share homology with Rce1, one of these proteins is likely an homolog of a second yeast CAAX protease, Ste24p.

FIGURE S2 Reconstitution of *cppB* in the CAAX prenyl-protease double-deletion mutant Δ*cppB*/Δ*cppA* restores the wild type phenotype. **(A)** Colony morphology of the complement strain Δ*cppB*/Δ*cppA+cppB* in comparison to the control strain Δ*akuB-pyrG^+^* and the Δ*cppB*/Δ*cppA* deletion mutant. **(B)** Colony diameter of Δ*cppB*/Δ*cppA+cppB* complement strain in comparison to the Δ*cppB*/Δ*cppA* deletion mutant and the control strain Δ*akuB-pyrG^+^*. Conidia (10^4^) of each strain were spotted on the middle of YPD agar plates. The plates were incubated at 37°C and the colony diameter was measured daily for 5 days. Measurements represent the average diameter of three independent experiments for each strain at the indicated timepoint. Error bars represent the standard deviation of the three independent experiments for each strain at the indicated timepoint. Statistics were performed using the Student’s *T*-test two-sample assuming equal variances and denote the recovery of growth in the complement strain when compared to the double mutant. **(C)** Conidial germination rates of the Δ*cppB*/Δ*cppA+cppB* complement strain in comparison to the Δ*cppB*/Δ*cppA* deletion mutant and the control strain Δ*akuB-pyrG^+^*. GMM broth was inoculated with conidia of each strain at a final concentration of 10^5^ conidia/ml and incubated at 37°C until the indicated timepoint. Polarity establishment was presented as the percentage of conidia forming a germtube from the total number of counted conidia for each strain at the indicated timepoint. Measurements and error bars represent the average and standard deviation of 3 independent experiments. Statistics were performed using the Student’s *T*-test two-sample assuming equal variances.

FIGURE S3 Quantitation of pixel intensity across hyphal segments of GFP-RasA, GFP-RasA^C206S^, and GFP-RasA^C207S^ expressing strains. Pixel intensity profiling capabilities of the Nikon Advanced Research software package were utilized to generate graphs depicting GFP-RasA protein distribution in cross-sections of hyphae. Three replicates are shown for each strain and hyphal cross-sections were chosen randomly within each microscopic field. Graphs for Replicate 1 from each strain were generated using the cross-sections depicted in **Figure 6**. For each graph, the x-axis is μm of hyphal width and the y-axis is pixel intensity. RasA presence at the cell-periphery (inner leaflet of the plasma membrane) is indicated by the peaks present on the outer portions of the GFP-RasA graphs generated for the Control Strain. The valley between represents lack of RasA presence in the cytoplasm or on internal membranes. Increased accumulation of RasA in the hyphal interior is evident upon mutation of the C207 residue and is further exacerbated by loss of CAAX proteolysis.

FIGURE S4 Minimal RasA plasma membrane localization is required for normal growth and development. Colony morphology of the control strain Δ*akuB-pyrG^+^* and the Δ*cppB*/Δ*cppA* mutant expressing RasA, RasA^C206S^, and RasA^C207S^ under the control of the *rasA* endogenous promoter. For each strain, 5000 conidia were spotted onto the middle of GMM agar plates and allowed to grow for 80 h at 37°C.
