# Supplementary figures and images for: C-terminus Proteolysis and Palmitoylation Cooperate for Optimal Plasma Membrane Localization of RasA in Aspergillus fumigatus
Source: Front Microbiol. 2018 Mar 26;9:562. doi: 10.3389/fmicb.2018.00562 (PMC5879109; doi:10.3389/fmicb.2018.00562)

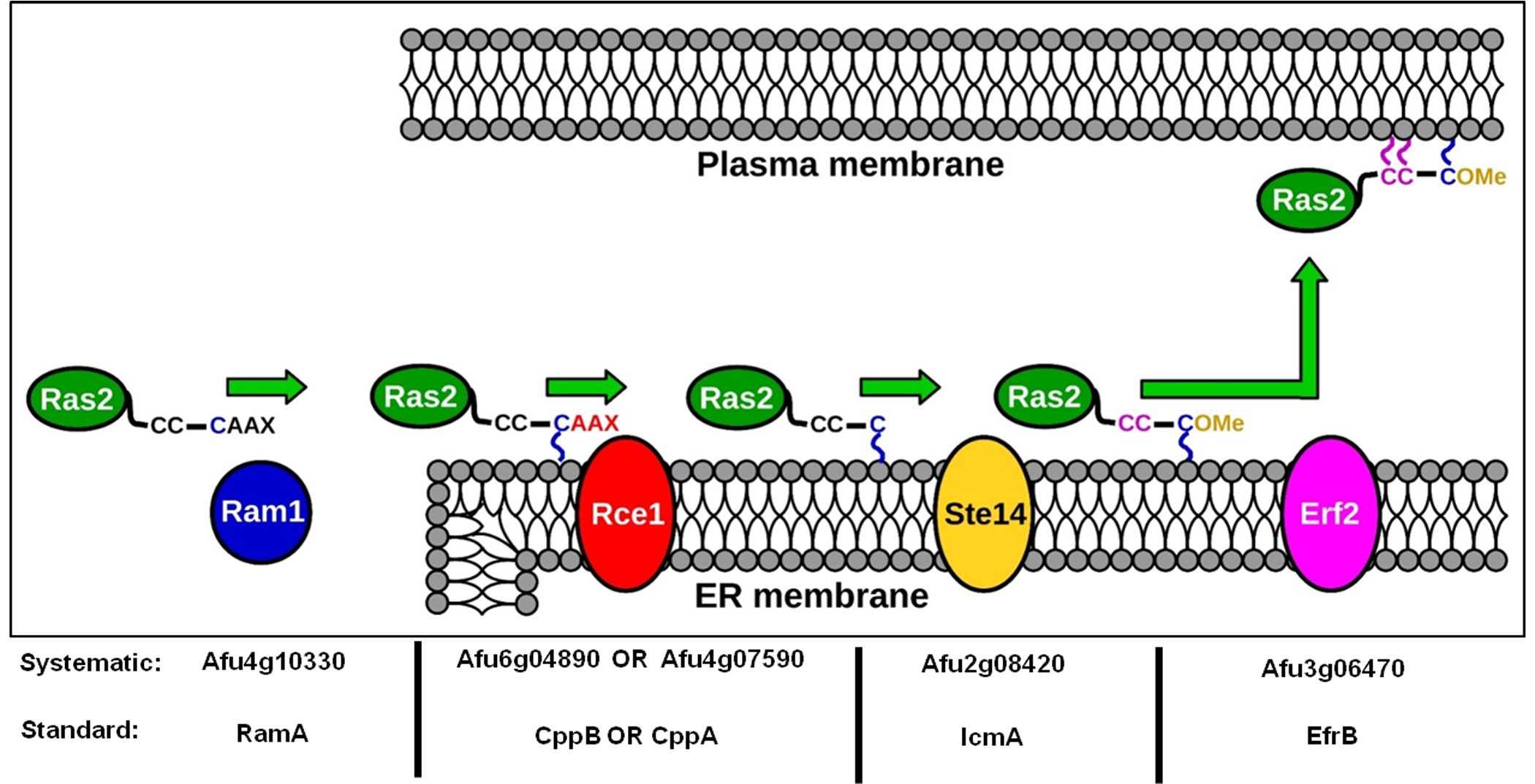

Supplement: FIGURE S1 — The post-prenylation CAAX maturation pathway. Shown is the previously characterized post-prenylation CAAX maturation pathway in Saccharomyces cerevisiae, with the RasA homolog, Ras2, as the substrate protein. Nascent Ras2 is a cytoplasmic protein that is prenylated by a prenyltransferase complex, typically farnesyltransferase, that is represented by it’s alpha-subunit, Ram1. After prenylation, Ras2 becomes associated with the endoplasmic reticulum (ER) membrane where the CAAX motif is proteolyzed by Rce1 (removing the –AAX residues) and subsequently methylated by the isoprenylcysteine-methyltransferase, Ste14. As a final step before trafficking to the plasma membrane, Ras2 is palmitoylated by a palmitoyltransferase complex (represented by Erf2) on a conserved cysteine residue upstream of the CAAX motif. This palmitoylation event provides greater hydrophobicity to the protein and drives Ras2 to the plasma membrane. The A. fumigatus homologs for each post-prenylation CAAX maturation component are shown below the figure. For Rce1, two potential homologs exist and have been named CppB and CppA. Although both share homology with Rce1, one of these proteins is likely an homolog of a second yeast CAAX protease, Ste24p. [file Image_1.TIF]

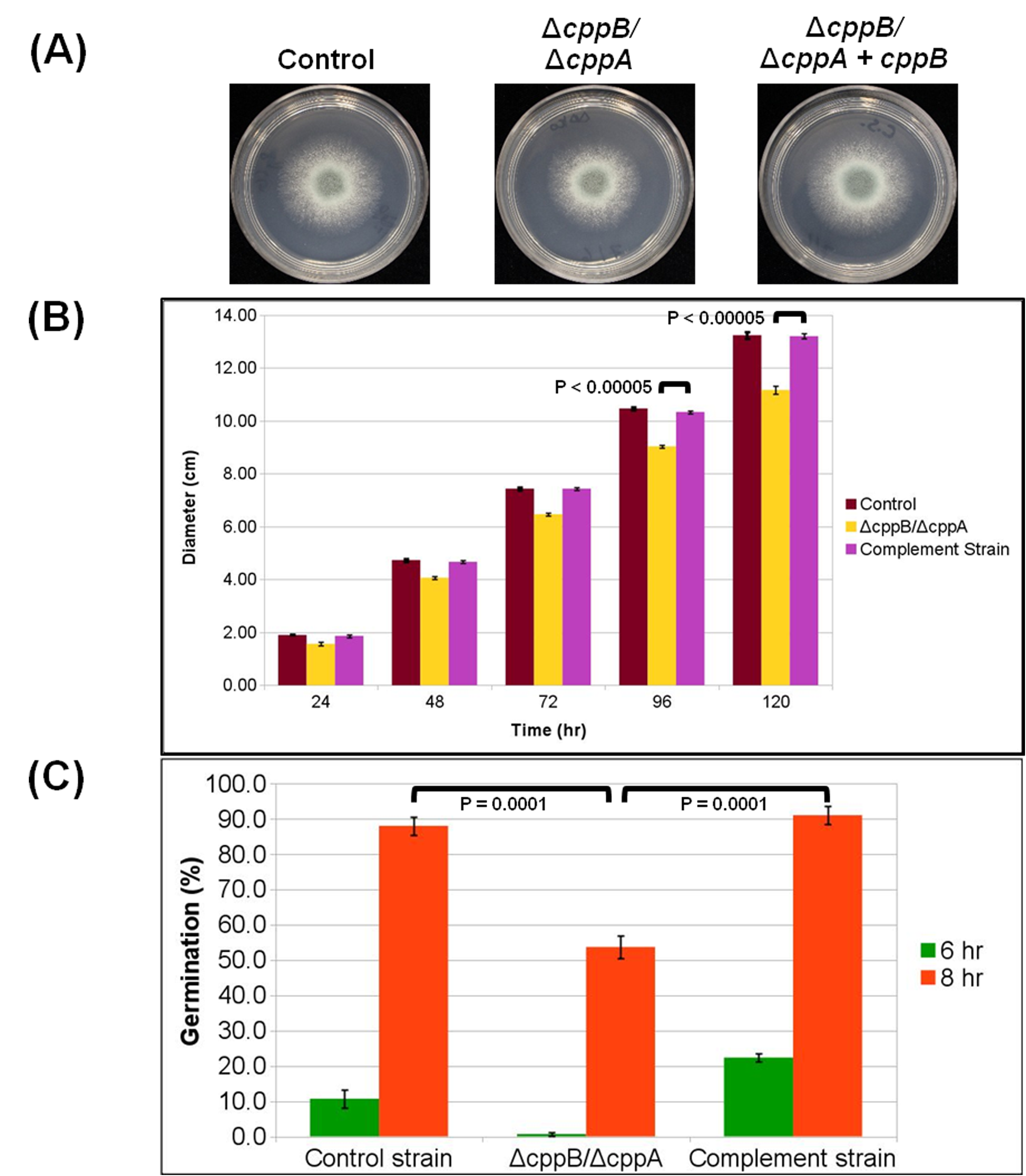

Supplement: FIGURE S2 — Reconstitution of cppB in the CAAX prenyl-protease double-deletion mutant ΔcppB/ΔcppA restores the wild type phenotype. (A) Colony morphology of the complement strain ΔcppB/ΔcppA+cppB in comparison to the control strain ΔakuB-pyrG+ and the ΔcppB/ΔcppA deletion mutant. (B) Colony diameter of ΔcppB/ΔcppA+cppB complement strain in comparison to the ΔcppB/ΔcppA deletion mutant and the control strain ΔakuB-pyrG+. Conidia (104) of each strain were spotted on the middle of YPD agar plates. The plates were incubated at 37°C and the colony diameter was measured daily for 5 days. Measurements represent the average diameter of three independent experiments for each strain at the indicated timepoint. Error bars represent the standard deviation of the three independent experiments for each strain at the indicated timepoint. Statistics were performed using the Student’s T-test two-sample assuming equal variances and denote the recovery of growth in the complement strain when compared to the double mutant. (C) Conidial germination rates of the ΔcppB/ΔcppA+cppB complement strain in comparison to the ΔcppB/ΔcppA deletion mutant and the control strain ΔakuB-pyrG+. GMM broth was inoculated with conidia of each strain at a final concentration of 105 conidia/ml and incubated at 37°C until the indicated timepoint. Polarity establishment was presented as the percentage of conidia forming a germtube from the total number of counted conidia for each strain at the indicated timepoint. Measurements and error bars represent the average and standard deviation of 3 independent experiments. Statistics were performed using the Student’s T-test two-sample assuming equal variances. [file Image_2.TIF]

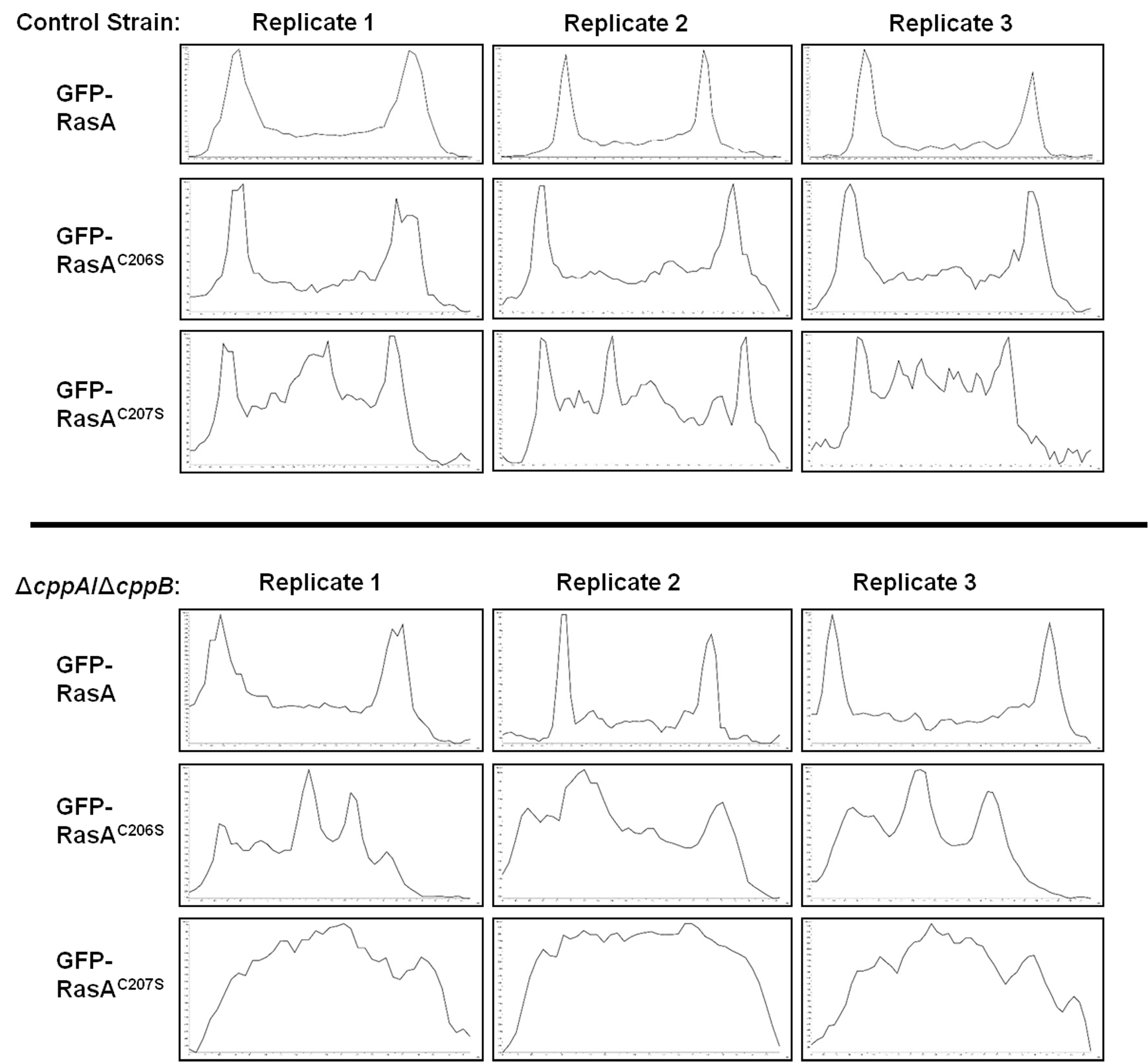

Supplement: FIGURE S3 — Quantitation of pixel intensity across hyphal segments of GFP-RasA, GFP-RasAC206S, and GFP-RasAC207S expressing strains. Pixel intensity profiling capabilities of the Nikon Advanced Research software package were utilized to generate graphs depicting GFP-RasA protein distribution in cross-sections of hyphae. Three replicates are shown for each strain and hyphal cross-sections were chosen randomly within each microscopic field. Graphs for Replicate 1 from each strain were generated using the cross-sections depicted in Figure 6. For each graph, the x-axis is μm of hyphal width and the y-axis is pixel intensity. RasA presence at the cell-periphery (inner leaflet of the plasma membrane) is indicated by the peaks present on the outer portions of the GFP-RasA graphs generated for the Control Strain. The valley between represents lack of RasA presence in the cytoplasm or on internal membranes. Increased accumulation of RasA in the hyphal interior is evident upon mutation of the C207 residue and is further exacerbated by loss of CAAX proteolysis. [file Image_3.TIF]

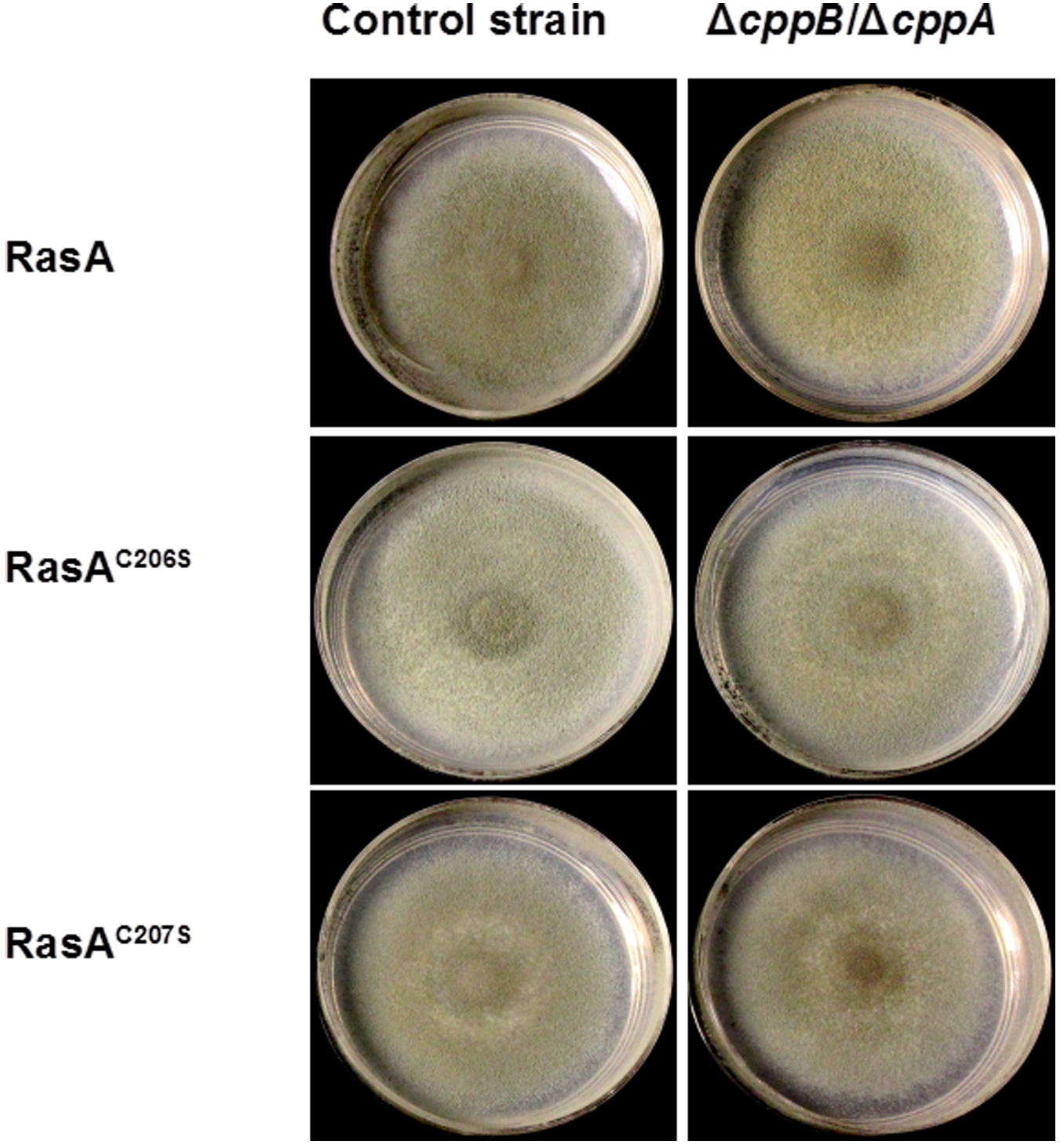

Supplement: FIGURE S4 — Minimal RasA plasma membrane localization is required for normal growth and development. Colony morphology of the control strain ΔakuB-pyrG+ and the ΔcppB/ΔcppA mutant expressing RasA, RasAC206S, and RasAC207S under the control of the rasA endogenous promoter. For each strain, 5000 conidia were spotted onto the middle of GMM agar plates and allowed to grow for 80 h at 37°C. [file Image_4.TIF]
